# Why Split Fees

**Published:** 1918-12

**Authors:** 


### Why Split Fees?

"I HEREBY DECLARE that during such time as I consider myself eligible to the privileges of the Department of Hospitals and Dispensaries I shall conform to the principle not to engage in the practice of the division of fees under any guise whatever. By this principle I understand that I am not to collect fees for others referring patients to me, nor to permit others to collect fees for me, nor to make joint fees with Physicians or Surgeons referring patients to me for operation or consultation, nor knowingly permit any agent or associate of mine to do so."

The above pledge is rightfully demanded of all appointees to the Attending Staff of the City institutions by the Department of Hospitals and Dispensaries of the City of Buffalo.

On the one hand this pledge should fill our hearts with shame as it lays bare a canker from which our profession is still suffering to a very large extent; on the other hand it reveals an economic wrong, an injustice to the hard working and underpaid general practitioner, which he has tried to remedy by the debasing and dishonest practice of the division of fees.

Without going into causes for this deplorable condition, which has been done time and again by other and abler pens, let us take up this matter in an academic way and let us see if a self-respecting and honest division of fees can be found.

One of our correspondents tells us that he has solved this question to his own satisfaction and that of the surgeons and consultants whom he calls in for aid in difficult cases; and this appears to us to be such a simple way out of a seemingly difficult situation that we recommend it for the consideration of our readers.

Our correspondent tells us that for the last fifteen or twenty years he has permitted the surgeon or consultant to fix the charges to be made to his patient and to collect these, while he himself charged the patient not less than 20% of this fee, aside from his other charges for visits and office consultations before and after the operation or consultation, and to specify this on the account for "diagnosis and presence at operation."

He has never had any objections on the part of his patients who were quick in realizing that this mode of procedure safeguarded them against overcharging on account of clandestine fee-splitting.

The surgeons and consultants knew beforehand that they would have to collect their own fees and those for their assistants directly from the patient and that they could, therefore, use their own judgment in fixing these. It also relieved them of a situation which at times was very embarrassing, namely, that they had to employ the general practitioner as assistant or anesthetist, so as to justify before the patient's family the fee which they intended to give him much as they would give a tip to a waiter or a railroad porter, when it would have been ever so much easier for them and safer for the patient if they could have brought their own tried assistants. Sapienti sat.

The above is contributed by one of our subscribers who wishes to be anonymous. In the general principle involved, we agree with it. We do not concur with the suggestion that any additional charge should be reflected to one man because of the services of another but, on the other hand, we believe that all professional charges whether medical or surgical, for consultant or attendant, by general practitioner or specialist, should be determined at their actual value, and not by the a la carte system of so much per call or office visit. It is almost unavoidable that the skill and reputation of the practitioner and the means of the patient should be considered in a charge but we do not regard as ethical, a plan by which one man secures a fancy fee in cash and another a routine fee on indefinite and dubious credit for the same case. Neither do we believe that the mere fact that a case involves a consultant or joint treatment, necessarily adds to its importance or to the charges appropriate, though a case that really needs consultation is usually worth more in fees than one that requires only one attendant.

It should be clearly recognized that no charge should be made surreptitiously; that, on account of professional dignity rather than an infringement of business honesty, no charge should be made, surreptitiously or openly either to a brother practitioner for getting him business or to a patient for securing him proper medical attention..

A clear cut distinction should be made between referring a practically independent condition to another practitioner and the joint attendance of several practitioners on a case that is a unit, so far as the patient is concerned. In the former instance, as when a patient is referred to a dentist, oculist, etc., by a family physician or another specialist, whether the patient is or is not being treated at the same time by the one referring him, each attendant should fix his own fee independently and assume the responsibility of collecting it. In the latter instance, as when surgical, medical and laboratory skill is united against a given condition, no arrangement is ethical which gives any one branch of practice an essential advantage as to amount of fee or time of collection, though this does not imply that the charge per unit of time spent or visit should be identical. Neither should the mere fact that one attendant has a more intimate acquaintance with the patient, personally or professionally, place him at a disadvantage. For these reasons, we believe that the best cure for the evils of fee splitting is on the basis of similia similibus. In other words, charge an adequate but not extortionate fee for the whole professional attendance on a given case, collect it as a unit and split it honestly and openly. This was done when congress paid the bill for the attendance on the late President McKinley and we believe in other somewhat analogous cases, and without complaint of violation of the code of ethics. Why should not the same course be pursued when the patient or his family pay the bill? Fee splitting of this kind is entirely different from the secret extortion of a percentage for throwing work to a consultant or from the slipping of a tip voluntarily—not to mention future favors by the man who can charge a fancy price to the poor devil who cannot. It seems to us fairer than the addition of a charge simply because of the fact of a consultation. Whether any definite system of dividing the fee could be worked out or whether the division would have to be made in each instance according to circumstances and depending on mutual fairness, can not and need not now be considered. The main point is that both ethical (?) and unethical graft and undignified methods would be excluded.

